# Neural Correlates of Attention to Human-Made Sounds: An ERP Study

**DOI:** 10.1371/journal.pone.0165745

**Published:** 2016-10-31

**Authors:** Katherine Kuhl-Meltzoff Stavropoulos, Leslie J. Carver

**Affiliations:** 1 University of California Riverside, Riverside, California, United States of America; 2 University of California San Diego, San Diego, California, United States of America; Sun Yat-Sen University, CHINA

## Abstract

Previous neuroimaging and electrophysiological studies have suggested that human made sounds are processed differently from non-human made sounds. Multiple groups have suggested that voices might be processed as “special,” much like faces. Although previous literature has explored neural correlates of voice perception under varying task demands, few studies have examined electrophysiological correlates of attention while directly comparing human made and non-human made sounds. In the present study, we used event-related potentials (ERPs) to compare attention to human versus non-human made sounds in an oddball paradigm. ERP components of interest were the P300, and fronto-temporal positivity to voices (FTVP), which has been reported in previous investigations of voice versus non-voice stimuli. We found that participants who heard human made sounds as “target” or infrequent stimuli had significantly larger FTPV amplitude, shorter FTPV latency, and larger P300 amplitude than those who heard non-human sounds as “target” stimuli. Our results are in concordance with previous findings that human-made and non-human made sounds are processed differently, and expand upon previous literature by demonstrating increased attention to human versus non-human made sounds, even when the non-human made sounds are ones that require immediate attention in daily life (e.g. a car horn). Heightened attention to human-made sounds is important theoretically and has potential for application in tests of social interest in populations with autism.

## Introduction

Understanding how individuals process and categorize social stimuli is important both for normative and abnormal social function. Social stimuli appear to have a privileged status in perceptual processing. Classic literature in the 1950s and 1960s led to the claim that “speech is special” based on perceptual studies comparing speech and non-speech stimuli [1]. It has long been argued that faces are “special” and processed differently than other types of visual stimuli [2–4]. More recently, it has been proposed that voices (including speech as well as other sounds made by people) are like “auditory faces” and may also be processed differently than other auditory stimuli [5, 6].

Several studies have attempted to determine whether there is a specialized response to particular kinds of auditory stimuli. Specifically, studies have compared brain responses to sounds made by animate versus inanimate sources (e.g. [7–9]), human-made sounds versus mechanically produced sounds (e.g. [10]), and human versus environmental sounds (e.g. [11, 12]). We will review findings suggesting that: 1) human voices are processed as “special,” 2) all sounds made directly by humans (including non-speech sounds such as yawning) are processed as “special,” and 3) sounds made by humans (even indirectly via instruments) are processed as “special.”

### Human Voices as Special

Groups have previously [12] recorded event-related brain potential (ERP) responses to human versus environmental and bird sounds while participants were engaged in a pure tone detection task. The authors observed larger amplitudes in response to human vocalizations compared to the other categories beginning as early as 164ms and peaking around 200ms. This effect was particularly evident at frontocentral electrodes FC5 and FC6. The authors termed this response the “fronto-temporal positivity to voices” (FTPV), and suggested that human voices may be processed as “special” compared to other auditory stimuli.

Other groups [13] compared ERP responses to frequent (distractor) stimuli that were either produced by a singer or an instrument (brass, wind, or string), while participants were instructed to respond to an infrequent (target) piano tone. The authors found differences in the ERPs elicited by human voices between 260 and 380ms after stimulus presentation. This difference was most evident at anterior, rather than posterior sites as is frequently the case with studies that utilize an oddball paradigm. This suggests that the difference between voices and instruments is likely not a P300 response to task-relevant, novel stimuli. Instead, the results of [13] suggest that sounds produced by the human voice may be “special” to the extent that they elicit larger amplitude ERP responses than sounds produced by other means, even when they are entirely incidental to the task. The authors [13] label this effect the “voice specific response” (VSR), and they suggest that perhaps because of the biological importance of human vocalizations, something akin to the P3a—which is observed in fronto-central electrodes in response to infrequent non-target stimuli often referred to as “novels” [14, 15]—occurs even when voices are not infrequently presented novel stimuli. Later investigations by the same group further investigated the VSR and found that when participants were instructed to watch a silent film during the stimulus presentations, the voice specific response was no longer present. Similarly, when participants were instructed to respond to only long (500ms) stimuli, and ignore all others, the voice specific response was absent, suggesting that the voice specific response is modulated by attention and may, at minimum, require attention to the same modality as the presented stimuli. The authors suggest that the VSR represents attention allocation on the basis of significance, rather than novelty as observed in the P3a [16]. Although the scalp distribution of the FTPV and VSR is similar, with an anterior focus, the primary difference between the FTPV [12] and the VSR appears to be latency. The FTPV peaks early, before about 200ms after stimulus presentation, whereas the VSR peaks later, closer to 300ms after stimulus onset.

Differences between brain responses to voice versus non-voice stimuli have also been observed using fMRI [17–19]. [17] compared neural responses to noise, frequency-modulated tones, reserved speech, pseudowords, and words. Regions in the superior temporal sulcus were more activated to words, pseudowords, and reversed speech compared to frequency-modulated tones. Interestingly, no significant differences were observed between the word, pseudoword, and reversed speech conditions. The authors suggest that the superior temporal gyrus responds strongly to speech sounds regardless of type. Similarly, [18] found selective activation of bilateral superior temporal sulci in response to speech versus both: 1) sinusoid sounds matched for mean fundamental frequency of speech, and 2) white noise sounds matched for the amplitude envelope of speech sounds. When comparing sounds by songbirds, other animals, human voices, and musical instruments, [19] found human speech selective clusters on the middle portion of the superior temporal cortex (including superior temporal gyri and sulci). The authors also found some musical instrument selective clusters, but no areas selective for either songbirds or other animals.

Previous studies have also utilized Magnetoencephalography (MEG) technology to investigate differential brain responses for voice versus non-voice stimuli. The authors [20] used identical stimuli to [13, 16] (i.e., sung tones versus instruments at the same pitch) and found a significantly larger neural response to voice versus non-voice stimuli in the sustained field (SF) MEG component, which peaks 400ms after stimulus onset. Thus, [13, 16, 20] all found increased neural activity for voice versus non-voice stimuli between 300-400ms after onset.

### Sounds Made Directly by Humans as Special

Several research groups have investigated whether human vocalizations more generally (e.g. clearing throat, cough) may be processed preferentially compared to sounds made by non-human sources [10, 11, 21, 22]. fMRI Results from [10, 11] suggest that selective activation of areas in the superior temporal sulcus (STS) is observed when comparing vocal versus non-vocal sounds. Furthermore, though regions of the STS were activated most strongly to speech versus non-speech vocalizations, both elicited more activity in the STS compared to their scrambled counterparts, which maintain the perceptual characteristics of vocalizations but are not perceived as vocalization by listeners. These findings suggest that it might not be specific auditory properties of voiced stimuli that activate these areas, but rather the conscious perception of a sound as vocal versus non-vocal (for an in depth review of the voice perception literature, see [5, 23]). [22] recorded fMRI responses in the prefrontal cortex to human vocalizations, animal (nonhuman) vocalizations, and non-vocal sounds (including musical instruments and environmental sounds). Results suggest that areas of the left prefrontal cortex are involved in processing the human voice specifically, and are preferentially activated even when vocal sounds do not contain linguistic information (e.g. non-speech vocalizations).

[21] recorded MEG responses to 70 vocal and 70 non-vocal sounds. The vocal sounds consisted of both speech (e.g., vowels) and non-speech (e.g., yawn) sounds. The non-vocal sounds consisted of animal sounds (e.g., dog bark), natural sounds (e.g., rain), and artificial sounds (e.g., bell). The authors found a significantly larger brain response for vocal sounds that peaked around 230ms that was located bilaterally in the mid-anterior portion of the STS. This component is thought to be the MEG equivalent of the FTPV described by [12], and provides further evidence that vocalizations made by humans (even non-speech), elicit a larger neural response than non-human sounds.

### Sounds Classified by Source, each “Special”

[7] used fMRI to identify brain regions activated by human and animal actions (living sounds) versus mechanical and environmental actions (non-living). In order to de-confound the effect of “living” sounds with speech, the authors chose to only use human produced sounds that were not speech (e.g. blowing nose, applause). The authors found that sounds produced by living things (humans or animals) activated auditory and sensorimotor regions in humans, whereas sounds produced by non-living things activated visually sensitive areas of the cortex. When the authors split the sounds into further categories (e.g. sounds made by humans versus animals), they found that human-produced sounds produced activation in the bilateral posterior superior temporal sulci regardless of task demands. The authors conclude that dissociation of cortical networks for different sound sources (broad categories of living versus non-living, and further split into animal, human, mechanical and environmental), provides support for the hypothesis that real-world sounds are categorically organized within the cortex.

[24] investigated electrophysiologic responses to human and animal vocalizations, as well as to environmental sounds (including musical instruments). The authors found increased neural response to human versus animal vocalizations, but did not find significant differences in waveform morphology between human vocalizations and other environmental sounds (e.g. musical instruments). [24] questioned the conclusion that voices are special—rather, they suggested that sounds made by conspecifics, even if made by a tool like an instrument, rather than the conspecific’s vocal apparatus, garner more attention that those made by other species.

### Current Study

Taken together, the results of previous studies suggest that humans show specialized brain responses to sounds that are made by humans. Imaging studies suggest that this brain response may be localized to the superior temporal area, where electrophysiology studies have observed “voice-specific” event-related potential (ERP) components. However, it is important to recognize that not all results support the idea that all human-made sounds are special, nor support the idea that human-made vocalizations in particular are processed uniquely. To be specific, though multiple fMRI and MEG studies have studied neural responses to vocalizations (both speech and non-speech) versus environmental sounds, we are not aware of electrophysiology studies that have utilized human made sounds comprised of speech, non-speech vocalizations, and human-made sounds that are not vocal (e.g. clapping), comparing them to particularly salient environmental sounds (e.g. car horn, telephone ring). The aim of the current study was to provide further information about electrophysiological measures of attention in response to human-made sounds when compared to salient environmental sounds.

In the current study, we utilized sounds that were either made by a person (hand clapping, laughing, humming, and a woman’s voice saying, “hi baby”), or not (car horn, telephone ring, whistle blowing, and timer beeping). Although previous studies have shown components that look similar to a P300 response, few studies have employed a standard oddball paradigm and compared responses to human made versus non-human made sounds when both serve as the infrequent (target) stimulus. We chose to analyze the P300 component in the current study as P300 target amplitude is thought to broadly represent allocation of attentional resources [25], encoding of significance [26], and orienting [27, 28], and P300 latency is thought to index classification speed [26], particularly when tasks are relatively undemanding [25]. Given previous reports of the fronto-temporal positivity to voices and its relevance to investigations of whether the human voice is particularly salient [12], or whether all sounds made directly by humans are salient [21] we also analyzed that component.

## Methods

### Participants

Sixty-nine adult participants (18 males and 43 females, which reflects the distribution of our available subject pool population) were randomly assigned to one of two groups: human-made target (infrequent) or non-human made target (infrequent). All subjects were native English speakers with no history of developmental disabilities or psychiatric conditions. Participants were recruited through the University of California subject pool, and the current study was approved by the University of California, San Diego Institutional Review Board. All participants were over 18 years of age, and signed a consent form.

### Task

Participants were randomly assigned to one of two conditions (human made target group, and non-human made target group), and tested in an oddball paradigm. The stimuli consisted of 8 sounds (4 human made, and 4 non-human made). Participants in both groups heard 400 trials (80% frequent, 20% infrequent). Which sounds were frequent versus infrequent differed between groups, such that individuals in the human made target group heard the human made sounds at a frequency of 20%, and non-human made sounds at a frequency of 80%, and individuals in the non-human made target group heard non-human made sounds at a frequency of 20%, and human made sounds at a frequency of 80%. Thus, each of the 4 target (infrequent) sounds were heard 20 times, and each of the non-target (frequent) sounds were heard 80 times.

The stimuli were pseudo-randomized for each group separately, with no sound occurring more than twice in a row. Participants sat in a chair about 12 inches from a computer monitor. Sounds were played out of Harmon Kadron loudspeakers attached to the monitor at 65 dB SPL. Participants were told to press a mouse button in response to the infrequent (target) sounds, and to refrain from responding to the frequent (non-target) sounds. In order to make the task clear, participants completed a short practice run during which they heard all of the frequent and infrequent sounds in the proportions outlined above. After the practice, participants were informed of any errors they had made, and could ask questions about the task.

### EEG Recording and Processing

Participants wore a standard, fitted cap (Electrocap International, Eaton, OH) with electrodes placed according to the international 10–20 system. Continuous EEG was recorded with a NeuroScan 4.5 System (Compumedics, Charlotte, NC, USA) with a reference electrode at Cz and re-referenced offline to the average activity at left and right mastoids. ERPs were recorded at 33 scalp locations using silver/silver-chloride (Ag/AgCl) electrodes at standard sites (Pz, Fz, O1, O2, P3, P4, T3, T4, T5, T6, C3, C4, Cz, F3, F4, F7, F8, A1, A2) and additional sites (CPz, FCz, CP5, CP6, CP1, CP2, FC1, FC2, FC5, FC6, FP1, FP2, AF7, AF8). Electrode resistance was kept under 10kOhms. Continuous EEG was amplified with a low pass filter (70Hz), a directly coupled high pass filter (DC), and a notch filter (60Hz). The signal was digitized at a rate of 250 samples per second via an Analog-to-Digital converter. Eye movement artifacts and blinks were monitored via horizontal electrooculogram (EOG) placed at the outer canthi of each eye and vertical EOG placed above and below the left eye. The baseline period was -100 to 0ms. Data was epoched from -200 to 800ms. Trials were separated by an inter-trial interval that varied between 1800 and 2000ms in duration. Trials with incorrect behavioral responses or electrophysiological artifacts were excluded from the averages.

Artifacts were removed via a four step process. Initially, the first author visually inspected all data for drift exceeding +/-200 mV in all electrodes, high frequency noise visible in all electrodes larger than 100 μV, and all flatlined data. Following initial inspection, data was epoched and eyeblink artifacts were identified using individual component analysis (ICA). Individual components were inspected alongside epoched data, and blink components were removed. Next, we utilized a moving window peak-to-peak procedure in ERPlab [29]. We utilized a 200ms moving window, a 100ms window step, and a 100 mV voltage threshold.

P300 amplitude was analyzed as the mean amplitude between 200-500ms after target stimulus onset, at midline electrodes (Fz, Cz, Pz). Electrodes of interest and time windows were selected based on previous literature for the P300 [30–32] and FTPV [9, 12, 13, 16], respectively. Time windows were verified by visual inspection of grand averaged waveforms, as well as confirmed for single-subjects. Latency of the P300 was analyzed as the 50% fractional area latency between 200-500ms after stimulus onset, at midline electrodes (Fz, Cz, Pz). Fractional peak latency is calculated by finding peak amplitude and working backwards until the point in the waveform at which the amplitude is a specific percentage of the peak value; in this case we used 50% [33]. The 50% value is commonly used and thought to be most reliable [33]. The FTPV was analyzed as the mean amplitude between 180-280ms after target stimulus onset, at electrodes FC5 and FC6. FTPV latency was analyzed as the 50% fractional area latency between 180-280ms after stimulus onset at electrodes FC5 and FC6.

### Stimuli

**Fig 1:**
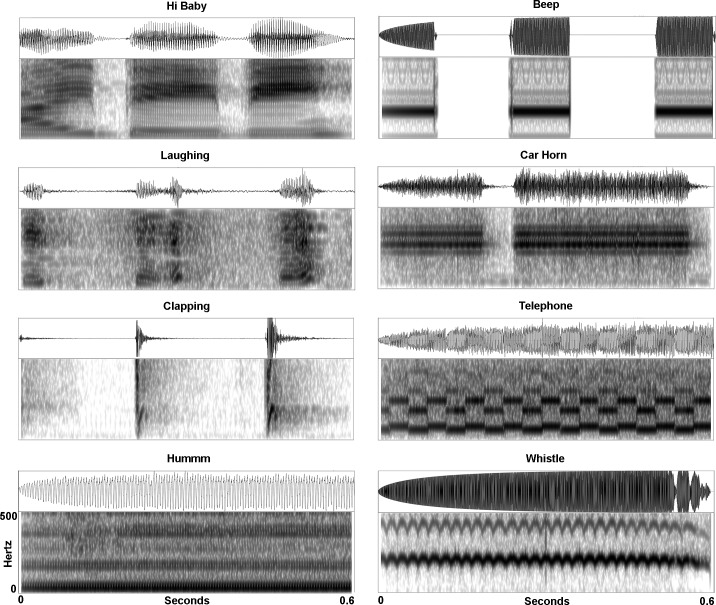
Spectrograms of sounds utilized in the current study.

Stimuli consisted of four human-made (defined here as sounds produced by a human voice or body) and four non-human made (defined here as sounds produced by a machine) sounds. The human-made sounds were the following: hands clapping, a woman humming a fixed tone, a woman saying “hi baby,” and a child laughing. The non-human-made sounds were the following: a car horn, a whistle blowing, a timer beeping, and a telephone ringing. All stimuli were naturally produced and instantly recognizable, and this was the intent in our study. Consequently, these 8 natural stimuli differed in their frequency and temporal characteristics. Importantly, however, using Adobe Audition, we controlled the stimuli for duration and RMS power: duration (*M* = 573ms, *SD* = 15.1ms for human made sounds, *M* = 588ms, *SD* = 6ms for non-human made sounds), average RMS power (*M* = -22.98dB, *SD* = .99dB for human made sounds, *M* = -22.24, *SD* = .69 for non-human made sounds, RMS power during the initial 300ms (*M* = -23.33dB, *SD* = 2.11 for human made sounds, *M* = -20.77dB, *SD* = 1.60 for non-human made sounds). Additionally, all stimuli had a smooth onset envelope (using Adobe Audition) to control for onset effects. Spectrograms of the individual sounds are shown in Fig 1.

In order to confirm that differences in familiarity between sounds would not contribute to our findings, a separate group of UCSD undergraduates (N = 19, 5 male, *M* = 21.46 years, *SD* = 1.67 years) listened to our stimuli in random order and were asked to correctly identify the sound from a list with all the sounds (8 total), and rate it for familiarity on a scale of 1–10. There were no differences between human-made and non-human made sounds on accuracy, *t*(75) = 1.34 *p* = .181, *n*.*s*, or in familiarity ratings *t*(67) = .917, *p* = .362, *n*.*s*.

### Analysis

All analyses were conducted using JMP (version 11.0). Mixed effect models were utilized for all analyses, with electrode as a within subjects variable, and group as a between subjects variable. Both amplitude and latency of the ERP components of interest were measured using target sounds only for each group. Therefore, level of “sound” was nested within the group variable, as the two groups had different “target” sounds. For final analysis, participants who made errors at a rate of 2 standard deviations above the mean (see below for mean errors) were rejected due to inattention (n = 5). Three participants were excluded due to computer error. Thus, our final sample and analysis included 31 participants in the human-made target group (10 male, *M* = 23.23 years, *SD* = 6.03), and 30 in the non-human made target group (8 male, *M* = 21.25 years, *SD* = 1.93).

## Results

The average number of errors for the human-made target and non-human made target groups were both under 1% (*M* = .83 errors out of 400 trials, *SD* = 2.17; *M* = 2.43 errors out of 400 trials, *SD* = 3.33) respectively. Neither the number of accepted target trials nor the number of accepted non-target trials differed between groups (all *p*s >.17). Age was not significantly different between groups (*p* = .09). However, due to the marginal effect of age between groups, we explored the relationship between age and ERP amplitude. Correlations were run between age and ERP amplitude—no relationship was observed (P300: *p* = .93, FTPV: *p* = .26).

### FTPV

Our analysis revealed a significant effect of group *F*(1, 59) = 7.89, *p* = .006, such that the human made target group had a significantly larger FTPV compared to the non-human made target group. A significant main effect of electrode was observed *F*(1, 242) = 9.28, *p* = .002, such that the FTPV was significantly larger at FC5 versus FC6. A significant interaction between electrode and group was observed, *F*(1, 242) = 18.91, *p* < .0001. Follow-up pairwise comparisons (bonferroni corrected) revealed a significant effect of electrode for the non-human made target group, *F*(1, 242) = 26.91, *p* < .0001 such that electrode FC5 had a significantly larger FTPV than FC6, but no significant effect of electrode for the human made target group.

**Fig 2:**
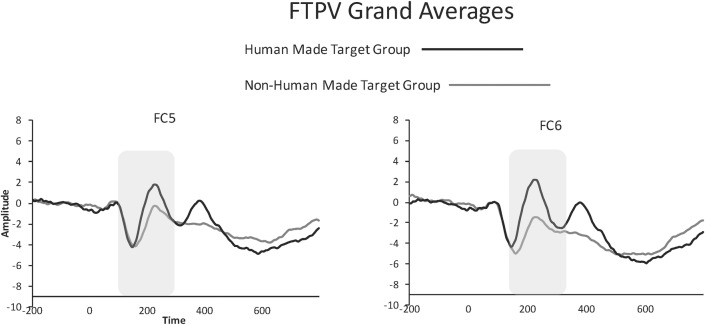
Grand averaged FTPV waveforms from electrodes FC5 and FC6 for both groups. Human made target group is shown in black, and non-human made target group in grey.

**Fig 3:**
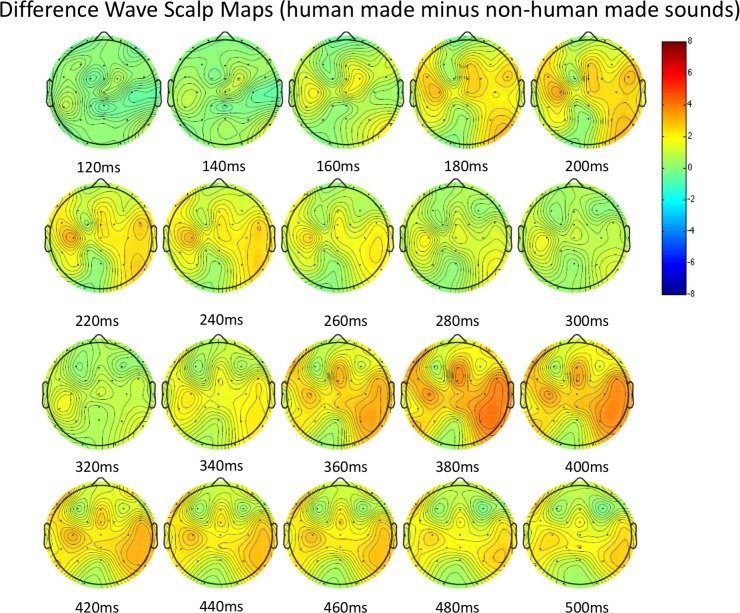
Scalp maps of difference wave (human made minus non-human made sounds) between 120-500ms.

These group effects were qualified by a main effect of sound, *F*(6, 177) = 6.5, *p* < .0001. Follow-up (bonferroni corrected) comparisons revealed that this effect was driven by a highly significant effect of sound within the human made target group (*p* < .0001). No sound effect was found in the non-human made target group (*p* = .83). Tukey’s HSD tests revealed that the “hi baby” stimulus elicited a significantly larger FTVP than clapping (*p* = .0002), and humming (*p* < .0001), but was not significantly different than laughing (*p* = .19). Laughing was not significantly different from clapping, but was significantly larger than humming (*p* = .03). Tukey’s HSD tests further revealed that in comparing sounds between groups, the “hi baby” stimulus was the only human made sound that was significantly different from each of the non human made sounds (all *p*s > .001), though laughing elicited a marginally larger FTPV than both car horn and beeping (*p*s = .07). Grand averaged waveforms for both groups are shown in Fig 2. Scalp maps depicting amplitude of a calculated difference wave (human-made minus non-human made sounds) each 20ms between 120-500ms after stimulus presentation are shown in Fig 3.

### FTPV Latency

Our analysis revealed a significant effect of group, *F*(1, 58.91) = 14.47, *p* = .0003, such that the human made target group had a significantly earlier FTPV versus the non-human made target group. A significant main effect of sound was also observed, *F*(6,165.4) = 2.68, *p* = .016. Follow-up (bonferroni corrected) pairwise comparisons revealed that this main effect was driven by significant differences between sounds within the human made target group (*p* = .0039). No sound effect was observed within the non-human made target group (*p* = .52). Within the human-made target group Tukey’s HSD tests revealed that laughing elicited a significantly earlier FTPV compared to humming (*p* = .02). Between groups, Tukey’s HSD tests revealed that both laughing and “hi baby” elicited significantly earlier FTPV responses than telephone, whistle, and car horn (all *p*s > .02). Neither laughing nor “hi baby” were significantly different than beeping.

### P300 Amplitude

A main effect of group was observed, *F*(1,58.02) = 22.88, *p* < .0001, such that the human made target group had significantly larger P300 than the non-human made target group. A significant effect of electrode was observed, *F*(2,464) = 22.67, *p* < .0001, such that the P300 was largest at electrode Pz and smallest at electrode Fz regardless of group assignment. This main effect of electrode was qualified by a significant interaction between electrode and group, *F*(2,464) = 41.74, *p* < .0001.

**Fig 4:**
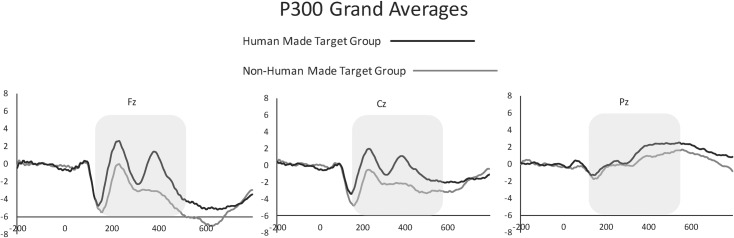
Grand averaged P300 waveforms from midline electrodes for both groups. Human made target group is shown in black, and non-human made target group in grey.

**Fig 5:**
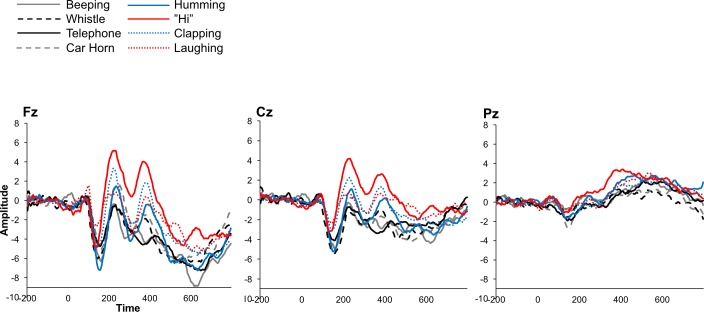
Grand averaged P300 waveforms for all sounds from midline electrodes.

In order to further analyze the differences between electrodes for each group, 2 ANOVAs were conducted for each group, a 4 (sound) by 3 (electrode) analysis. Within the non-human made target group, a main effect of electrode was observed, *F*(2,58) = 10.37, *p* = .0001, such that the P300 was largest at electrode Pz. Within the human made target group, a significant effect of electrode was observed, *F*(2,58.04) = 7.32, *p* = .001, such that the P300 was largest at electrode Fz and smallest at electrode Pz. No other significant main effects or interactions were observed. Grand averaged waveforms from both groups are shown in Fig 4. Grand average waveforms of each individual sound from each group are shown in Fig 5.

### P300 Latency

A main effect of electrode was found, *F*(2,55.56), *p* < .0001, such that the P300 occurred latest at electrode Pz. No other main effects or interactions were observed.

## Discussion

We process sounds made by conspecifics as special, and attend to them as though they are unique. Some studies have suggested that human made sounds are important because they carry social information [5, 23], whereas others have suggested that any sound that signals the need for immediate action might be given priority [24]. In the present study we used an oddball paradigm in which we presented human made and non-human made sounds to elicit two brain components of interest—one peaking around 250ms, and another peaking around 380ms.

By using four different human made and four non-human made sounds that are all encountered often, we hoped to clarify whether environmental importance alone (e.g., one must immediately respond to a car horn), could account for differences observed in previous studies that compared responses to human made and non-human made sounds. We hypothesized that an oddball paradigm might elicit the VSR and/or FTPV described by other authors [12, 13, 16]. We sought to expand upon results from those groups, and understand whether differing ERP responses to human-made stimuli would occur under different task requirements, even when not all sounds were voiced. We were particularly interested in previous authors’ evidence that increased ERP responses to voiced stimuli might not be voice-specific, but might also be sensitive to other human made sounds (e.g. coughing, clearing throat, [12]). We hoped to replicate these results, as well as clarify whether other human made sounds that were not vocalized (e.g., clapping), might also elicit this component. We hypothesized that human made sounds would elicit a larger ERP response than non-human made environmental sounds because of their social importance. Our findings largely support this hypothesis, and suggest that human-made sounds elicit a larger ERP response in adults versus non-human made sounds, even if the non-human made sounds are highly salient.

### FTPV

We found that target human made sounds elicited a larger ERP response than target non-human made sounds. The topographic distribution and time course of this activation is consistent with the FTPV described by [12]. [12] observed the increased ERP response to voice vs. environmental or bird sounds at fronto-temporal electrode sites peaking at 200ms while participants engaged in a pure tone detection task. The authors found that while speech stimuli contributed strongly to the FTPV, other non-speech vocalizations elicited a FTPV. Results of the current study suggest that although overall the FTPV is larger and occurs earlier in response to human made versus non-human made sounds, both FTPV amplitude and latency differ in response to individual sounds. The only human made sound that was significantly larger in amplitude from all non human made sounds was a woman saying, “hi baby.” Similarly, though human made sounds elicited an earlier FTPV than non human made sounds, laughing and “hi baby” were the only two human made sounds that were significantly different from three of the four the non-human made sounds (laughing and “hi baby” were not significantly faster than beeping). Taken together, our findings are in agreement with those of [12] in that human vocalizations appear to elicit the largest and earliest FTPV response.

Although the morphology and time course of our early component is consistent with the FTPV, it is important to note that we used a markedly different task than [12]. In the present study, participants were asked to listen to sounds that were 500ms in duration, to press a button when they heard the target or infrequent sounds, and to inhibit responses to the nontarget (frequent) sounds. In contrast, [12] had participants listening to sounds of 200ms duration while simultaneously engaging in a pure tone detection task. Thus, [12] was directly comparing non-target stimuli (e.g., voices, bird song, and environmental sounds that were incidental to their task), while our task directly compared human-made versus non-human made sounds as target stimuli.

### P300

The topographic distribution and time course of our later component is consistent with a P300 response, as well as the voice specific response (VSR) described by [13, 16]. However, [13, 16] used wind, brass, and string instruments as well as voices as incidental non-target stimuli while participants were instructed to listen for and respond to a piano tone. Thus, the comparison of interest in previous studies was between different types of distractor sounds, which differs from the current study. Although the present study utilized different stimuli, as well as different task requirements from [13, 16], our results have interesting implications for the VSR. Our results suggest that the VSR may be less of a voice specific response, and more of a “human-made sound” response, as we found a significant effect of group (human target versus non-human target) that was not qualified by an effect of sound. Our results are consistent with studies that suggest that human made sounds are treated with special importance as suggested by [24]. [24] found nominal differences suggesting that human vocalizations elicit more neural activity compared to other sound types. However, these differences were only significant when human vocalizations were compared to animal vocalizations. Differences in stimuli, as well as task demands, may explain the differences in results.

Of particular interest is that the topographic distribution observed in the current study is consistent with the VSR for the human made target group (e.g., largest at frontal sites), but consistent with previous P300 literature for the non-human made target group (e.g., largest at parietal sites). As mentioned by [13, 16], the VSR is similar in topography and timing to the P3a, or Novelty P3 component, which is thought to be elicited to outstanding distractors within an odd-ball paradigm or stimuli that require immediate attention regardless of their task relevance, and is thought to reflect vigilance [15]. Thus, we cannot discount the possibility that within the current study we observed a VSR response (albeit for a set of stimuli that were expanded to non-voiced human made sounds) for the human made target group, and a P300 response for the non-human target group. That is to say, it is possible that we observed a VSR response for human-made target sounds, and a more “typical” P300 response for non-human made target sounds.

### Limitations

The current study has limitations that should be addressed. Although we controlled our auditory stimuli for a variety of factors (e.g. RMS power, duration, and initial onset envelope), the sounds are not identical with regard to frequency characteristics and temporal variations. Thus, the possibility of effects due to acoustic differences in stimuli is not eliminated. However, our goal was to compare “natural” sounds made by humans as opposed to those not made by humans, and we intentionally used multiple instances of each category to focus on the category differences. Our study was designed to understand whether differences in ERP responses could be observed between human and non-human made sounds that were familiar and often encountered, and this goal was achieved.

## Conclusions

The present study provides information about the neural underpinnings of processing human made versus non-human made sounds. Our results suggest that these two types of sounds are processed differently in the brain—even when the non-human made sounds are considered environmentally salient. Future studies could utilize oddball paradigms to differentiate between ERP components to voiced/vocal but not voiced (e.g., cough), and non-vocal human-made stimuli (e.g., clapping). Another useful direction would be to use this paradigm to investigate whether differences in these ERP components are observed in adults with disorders related to social attention (e.g., autism spectrum disorder), as this might provide useful information about the neural underpinnings of social processing deficits.

## References

[1] Liberman AM, Cooper FS, Shankweiler DP, Studdert-Kennedy M. Perception of the speech code. Psychol Rev. 1967;74(6):431–61. . 4170865 10.1037/h0020279

[2] Kanwisher N, McDermott J, Chun MM. The fusiform face area: a module in human extrastriate cortex specialized for face perception. J Neurosci. 1997;17(11):4302–11. . 9151747 10.1523/JNEUROSCI.17-11-04302.1997 PMC6573547

[3] McCarthy G, Puce A, Belger A, Allison T. Electrophysiological studies of human face perception. II: Response properties of face-specific potentials generated in occipitotemporal cortex. Cereb Cortex. 1999;9(5):431–44. . 10450889 10.1093/cercor/9.5.431

[4] Puce A, Allison T, Gore JC, McCarthy G. Face-sensitive regions in human extrastriate cortex studied by functional MRI. J Neurophysiol. 1995;74(3):1192–9. . 7500143 10.1152/jn.1995.74.3.1192

[5] Belin P, Fecteau S, Bedard C. Thinking the voice: neural correlates of voice perception. Trends Cogn Sci. 2004;8(3):129–35. 10.1016/j.tics.2004.01.008 . 15301753

[6] Belizaire G, Fillon-Bilodeau S, Chartrand JP, Bertrand-Gauvin C, Belin P. Cerebral response to ‘voiceness’: a functional magnetic resonance imaging study. NeuroReport. 2006;18:29–33. 10.1097/WNR.0b013e3280122718 17259856

[7] Engel LR, Frum C, Puce A, Walker NA, Lewis JW. Different categories of living and non-living sound-sources activate distinct cortical networks. Neuroimage. 2009;47(4):1778–91. 10.1016/j.neuroimage.2009.05.041 19465134 PMC2774089

[8] Lewis JW, Brefczynski JA, Phinney RE, Janik JJ, DeYoe EA. Distinct cortical pathways for processing tool versus animal sounds. J Neurosci. 2005;25(21):5148–58. 10.1523/JNEUROSCI.0419-05.2005 . 15917455 PMC6724809

[9] Murray MM, Camen C, Gonzalez Andino SL, Bovet P, Clarke S. Rapid brain discrimination of sounds of objects. J Neurosci. 2006;26(4):1293–302. 10.1523/JNEUROSCI.4511-05.2006 . 16436617 PMC6674563

[10] Belin P, Zatorre RJ, Ahad P. Human temporal-lobe response to vocal sounds Cognitive Brain Research. 2002;13:17–26. 11867247 10.1016/s0926-6410(01)00084-2

[11] Belin P, Zatorre RJ, Lafallle P, Ahad P, Pike B. Voice-selective areas in human auditory cortex. Nature 2000;403:309–12. 10.1038/35002078 10659849

[12] Charest I, Pernet CR, Rousselet GA, Quinones I, Latinus M, Fillion-Bilodeau S, et al Electrophysiological evidence for an early processing of human voices. BMC Neurosci. 2009;10:127 10.1186/1471-2202-10-127 19843323 PMC2770575

[13] Levy DA, Granot R, Bentin S. Processing specificity for human voice stimuli: electrophysiological evidence. Neuroreport. 2001;12(12):2653–7. . 11522942 10.1097/00001756-200108280-00013

[14] Fabiani M, Friedman D. Changes in brain activity patterns in aging: the novelty oddball. Psychophysiology. 1995;32(6):579–94. . 8524992 10.1111/j.1469-8986.1995.tb01234.x

[15] Friedman D, Cycowicz YM, Gaeta H. The novelty P3: an event-related brain potential (ERP) sign of the brain's evaluation of novelty. Neurosci Biobehav Rev. 2001;25(4):355–73. . 11445140 10.1016/s0149-7634(01)00019-7

[16] Levy DA, Granot R, Bentin S. Neural sensitivity to human voices: ERP evidence of task and attentional influences. Psychophysiology. 2003;40(2):291–305. . 12820870 10.1111/1469-8986.00031

[17] Binder JR, Frost JA, Hammeke TA, Bellgowan PS, Springer JA, Kaufman JN, et al Human temporal lobe activation by speech and nonspeech sounds. Cereb Cortex. 2000;10(5):512–28. . 10847601 10.1093/cercor/10.5.512

[18] Grandjean D, Sander D, Pourtois G, Schwartz S, Seghier ML, Scherer KR, et al The voices of wrath: brain responses to angry prosody in meaningless speech. Nat Neurosci. 2005;8(2):145–6. 10.1038/nn1392 . 15665880

[19] Leaver AM, Rauschecker JP. Cortical representation of natural complex sounds: effects of acoustic features and auditory object category. J Neurosci. 2010;30(22):7604–12. 10.1523/JNEUROSCI.0296-10.2010 20519535 PMC2930617

[20] Gunji A, Koyama S, Ishii R, Levy D, Okamoto H, Kakigi R, et al Magnetoencephalographic study of the cortical activity elicited by human voice. Neurosci Lett. 2003;348(1):13–6. . 12893414 10.1016/s0304-3940(03)00640-2

[21] Capilla A, Belin P, Gross J. The early spatio-temporal correlates and task independence of cerebral voice processing studied with MEG. Cereb Cortex. 2013;23(6):1388–95. 10.1093/cercor/bhs119 . 22610392

[22] Fecteau S, Armony JL, Joanette Y, Belin P. Sensitivity to voice in human prefrontal cortex. J Neurophysiol. 2005;94(3):2251–4. 10.1152/jn.00329.2005 . 15928057

[23] Belin P, Bestelmeyer PE, Latinus M, Watson R. Understanding voice perception. Br J Psychol. 2011;102(4):711–25. 10.1111/j.2044-8295.2011.02041.x . 21988380

[24] De Lucia M, Clarke S, Murray MM. A temporal hierarchy for conspecific vocalization discrimination in humans. J Neurosci. 2010;30(33):11210–21. 10.1523/JNEUROSCI.2239-10.2010 . 20720129 PMC6633490

[25] Polich J. Updating P300: an integrative theory of P3a and P3b. Clin Neurophysiol. 2007;118(10):2128–48. 10.1016/j.clinph.2007.04.019 17573239 PMC2715154

[26] Kutas M, McCarthy G, Donchin E. Augmenting mental chronometry: the P300 as a measure of stimulus evaluation time. Science. 1977;197(4305):792–5. . 887923 10.1126/science.887923

[27] Nieuwenhuis S, Aston-Jones G, Cohen JD. Decision making, the P3, and the locus coeruleus-norepinephrine system. Psychol Bull. 2005;131(4):510–32. 10.1037/0033-2909.131.4.510 . 16060800

[28] Nieuwenhuis S, De Geus EJ, Aston-Jones G. The anatomical and functional relationship between the P3 and autonomic components of the orienting response. Psychophysiology. 2011;48(2):162–75. 10.1111/j.1469-8986.2010.01057.x 20557480 PMC3797154

[29] Lopez-Calderon J, Luck SJ. ERPLAB: an open-source toolbox for the analysis of event-related potentials. Front Hum Neurosci. 2014;8:213 10.3389/fnhum.2014.00213 24782741 PMC3995046

[30] Bledowski C, Prvulovic D, Hoechstetter K, Scherg M, Wibral M, Goebel R, et al Localizing P300 generators in visual target and distractor processing: a combined event-related potential and functional magnetic resonance imaging study. J Neurosci. 2004;24(42):9353–60. 10.1523/JNEUROSCI.1897-04.2004 . 15496671 PMC6730097

[31] Ilan AB, Polich J. P300 and response time from a manual Stroop task. Clin Neurophysiol. 1999;110(2):367–73. . 10210626 10.1016/s0168-5597(98)00053-7

[32] Polich J. Normal variation of P300 from auditory stimuli. Electroencephalogr Clin Neurophysiol. 1986;65(3):236–40. . 2420577 10.1016/0168-5597(86)90059-6

[33] Luck SJ. An introduction to the event-related potential technique Cambridge, MA: The MIT Press; 2005.

